# Incorporating biological structure into machine learning models in biomedicine

**DOI:** 10.1016/j.copbio.2019.12.021

**Published:** 2020-01-18

**Authors:** Jake Crawford, Casey S Greene

**Affiliations:** 1 Graduate Group in Genomics and Computational Biology, Perelman School of Medicine, University of Pennsylvania, Philadelphia, PA, United States; 2 Department of Systems Pharmacology and Translational Therapeutics, Perelman School of Medicine, University of Pennsylvania, Philadelphia, PA, United States; 3 Childhood Cancer Data Lab, Alex’s Lemonade Stand Foundation, Philadelphia, PA, United States

## Abstract

In biomedical applications of machine learning, relevant information often has a rich structure that is not easily encoded as real-valued predictors. Examples of such data include DNA or RNA sequences, gene sets or pathways, gene interaction or coexpression networks, ontologies, and phylogenetic trees. We highlight recent examples of machine learning models that use structure to constrain model architecture or incorporate structured data into model training. For machine learning in biomedicine, where sample size is limited and model interpretability is crucial, incorporating prior knowledge in the form of structured data can be particularly useful. The area of research would benefit from performant open source implementations and independent benchmarking efforts.

## Introduction

It can be challenging to distinguish signal from noise in biomedical datasets, and machine learning methods are particularly hampered when the amount of available training data is small. Incorporating biomedical knowledge into machine learning models can reveal patterns in noisy data [1] and aid model interpretation [2]. Biological knowledge can take many forms, including genomic sequences, pathway databases, gene interaction networks, and knowledge hierarchies such as the Gene Ontology [3]. However, there is often no canonical way to encode these structures as real-valued predictors. Modelers must creatively decide how to encode biological knowledge that they expect will be relevant to the task.

Biomedical datasets often contain more input predictors than data samples [4,5]. A genetic study may genotype millions of single nucleotide polymorphisms (SNPs) in thousands of individuals, or a gene expression study may profile the expression of thousands of genes in tens of samples. Thus, it can be useful to include prior information describing relationships between predictors to inform the representation learned by the model. This contrasts with non-biological applications of machine learning, where one might fit a model on millions of images [6] or tens of thousands of documents [7], making inclusion of prior information unnecessary.

We review approaches that incorporate external information about the structure of desirable solutions to learn from biomedical data. One class of commonly used approaches learns a representation that considers the context of each base pair from raw sequence data. For models that operate on gene expression data or genetic variants, it can be useful to incorporate networks or pathways describing relationships between genes. We also consider other examples, such as neural network architectures that are constrained based on biological knowledge.

There are many complementary ways to incorporate heterogeneous sources of biomedical data into the learning process, which have been covered elsewhere [8,9]. These include feature extraction or representation learning before modeling and/or other data integration methods that do not necessarily involve customizing the model itself.

## Sequence models

**Figure 1:**
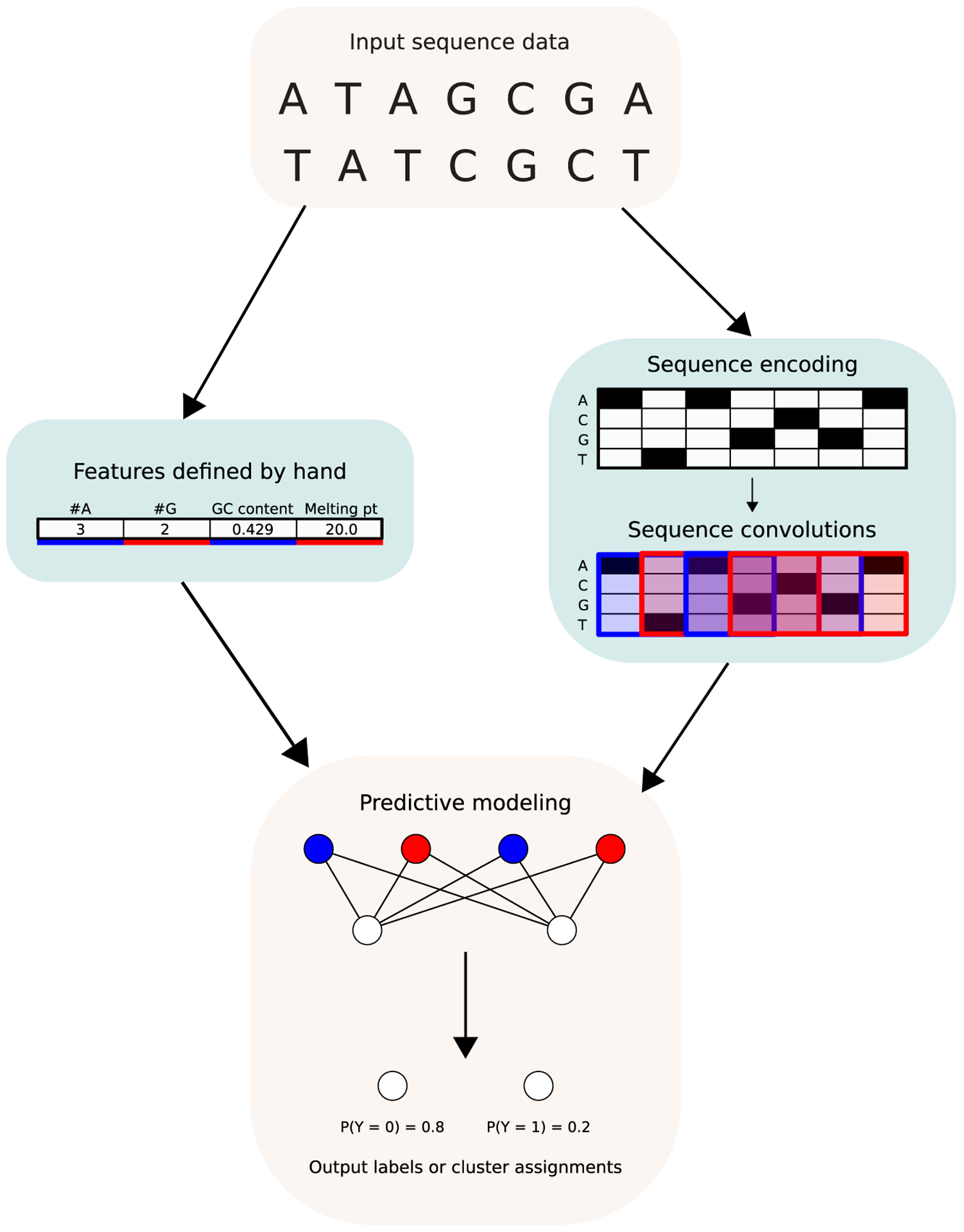
Contrasting approaches to extracting features from DNA or RNA sequence data. Early models defined features of interest by hand based on prior knowledge about the prediction or clustering problem of interest, such as GC content or sequence melting point, as depicted in the left branch in the figure. Convolutional models, depicted in the right branch, use sequence convolutions to derive features directly from sequence proximity, without requiring quantities of interest to be identified before the model is trained. Red or blue emphasis denotes inputs to the predictive model (either the hand-defined numeric features on the left or the outputs of convolutional filters on the right).

Early neural network models primarily used hand-engineered sequence features as input to a fully connected neural network [10,11] (Figure 1). As convolutional neural network (CNN) approaches matured for image processing and computer vision, researchers leveraged biological sequence proximity similarly. CNNs are a neural network variant that groups input data by spatial context to extract features for prediction.

The definition of ‘spatial context’ is specific to the input: one might group image pixels that are nearby in 2D space, or genomic base pairs that are nearby in the linear genome. In this way, CNNs consider context without making strong assumptions about exactly how much context is needed or how it should be encoded; the data inform the encoding. A detailed description of how CNNs are applied to sequences can be found in Angermueller *et al*. [12].

## Applications in regulatory biology

Many early applications of deep learning to biological sequences were in regulatory biology. Early CNNs for sequence data predicted binding protein sequence specificity from DNA or RNA sequence [13], variant effects from noncoding DNA sequence [14], and chromatin accessibility from DNA sequence [15].

Recent sequence models take advantage of hardware advances and methodological innovation to incorporate more sequence context and rely on fewer modeling assumptions. BPNet, a CNN that predicts transcription factor binding profiles from DNA sequences, accurately mapped known locations of binding motifs in mouse embryonic stem cells [16••]. BPNet considers 1000 base pairs of context around each position when predicting binding probabilities with a technique called dilated convolutions [17], which is particularly important because motif spacing and periodicity can influence binding. cDeepbind [18•] combines RNA sequences with information about secondary structure to predict RNA binding protein affinities. Its convolutional model acts on a feature vector combining sequence and structural information, using context for both to inform predictions. APARENT [19] is a CNN that predicts alternative polyadenylation (APA) from a training set of over three million synthetic APA reporter sequences. These diverse applications underscore the power of modern deep learning models to synthesize large sequence datasets.

Models that consider sequence context have also been applied to epigenetic data. DeepSignal [20] is a CNN that uses contextual electrical signals from Oxford Nanopore single-molecule sequencing data to predict 5mC or 6 mA DNA methylation status. MRCNN [21] uses sequences of length 400, centered at CpG sites, to predict 5mC methylation status. Deep learning models have also been used to predict gene expression from histone modifications [22,23•]. Here, a neural network model consisting of long short-term memory (LSTM) units was used to encode the long-distance interactions of histone marks in both the 3′ and 5′ genomic directions. In each of these cases, proximity in the linear genome helped model the complex interactions between DNA sequence and epigenome.

## Applications in variant calling and mutation detection

Identification of genetic variants also benefits from models that include sequence context. DeepVariant [24••] applies a CNN to images of sequence read pileups, using read data around each candidate variant to accurately distinguish true variants from sequencing errors. CNNs have also been applied to single molecule (PacBio and Oxford Nanopore) sequencing data [25], using a different sequence encoding that results in better performance than DeepVariant on single molecule data. However, many variant calling models still use hand-engineered sequence features as input to a classifier, including current state-of-the-art approaches to insertion/deletion calling [26,27]. Detection of somatic mutations is a distinct but related challenge to detection of germline variants, and has also recently benefitted from use of CNNs [28].

## Network-based and pathway-based models

**Figure 2:**
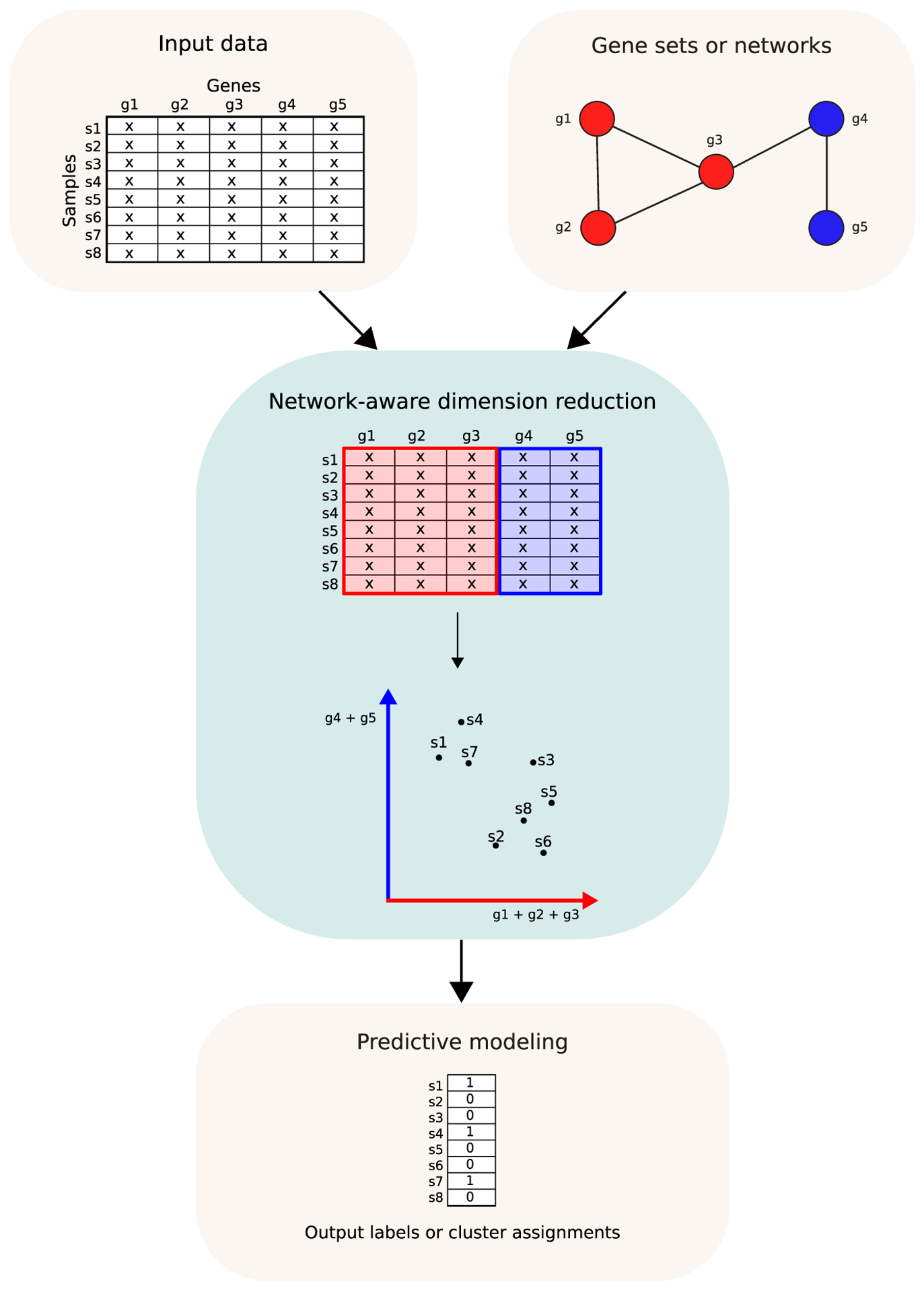
The relationships between genes provide structure that can be incorporated into machine learning models. One common approach is to use a network or collection of gene sets to embed the data in a lower-dimensional space, in which genes that are in the same gene sets or that are well-connected in the network have a similar representation in the lower-dimensional space. The embedded data can then be used for classification or clustering tasks. The ‘x’ values in the data table represent gene expression measurements.

Rather than operating on sequences, many machine learning models in biomedicine operate on inputs that lack intrinsic order. Models may make use of gene expression data matrices from RNA sequencing or micro-array experiments in which rows represent samples and columns represent genes. To account for relationships between genes, one might incorporate known interactions or correlations when making predictions or generating a low-dimensional representation of the data (Figure 2). This is comparable to the manner in which sequence context pushes models to consider nearby base pairs similarly.

## Applications in transcriptomics

Models built from gene expression data can benefit from incorporating gene-level relationships. One form that this knowledge commonly takes is a database of gene sets, which may represent biological pathways or gene signatures for a biological state of interest. PLIER [29••] uses gene set information from MSigDB [30] and cell type markers to extract a representation of gene expression data that corresponds to biological processes and reduces technical noise. The resulting gene set-aligned representation accurately decomposed cell type mixtures. MultiPLIER [31] applied PLIER to the recount2 gene expression compendium [32] to develop a model that shares information across multiple tissues and diseases, including rare diseases with limited sample sizes. PASNet [33] uses MSigDB to inform the structure of a neural network for predicting patient outcomes in glioblastoma multiforme (GBM) from gene expression data. This approach aids interpretation, as pathway nodes in the network with high weights can be inferred to correspond to certain pathways in GBM outcome prediction.

Gene-level relationships can also be represented with networks. Network nodes typically represent genes and real-valued edges may represent interactions or correlations between genes, often in a tissue or cell type context of interest. Network-based stratification [34] is an early example of a method for utilizing gene interaction network data in this manner, applied to improve subtyping across several cancer types. More recently, netNMF-sc [35••] incorporates coexpression networks [36] as a smoothing term for dimension reduction and dropout imputation in single-cell gene expression data. The coexpression network improves performance for identifying cell types and cell cycle marker genes, as compared to using raw gene expression or other single-cell dimension reduction methods. Combining gene expression data with a network-derived smoothing term also improved prediction of patient drug response in acute myeloid leukemia [37•] and detection of mutated cancer genes [38]. PIMKL [39•] combines network and pathway data to predict disease-free survival from breast cancer cohorts. This method takes as input both RNA-seq gene expression data and copy number alteration data, but can also be applied to gene expression data alone.

Gene regulatory networks can also augment models for gene expression data. These networks describe how the expression of genes is modulated by biological regulators such as transcription factors, microRNAs, or small molecules. creNET [40••] integrates a gene regulatory network, derived from STRING [41], with a sparse logistic regression model to predict phenotypic response in clinical trials for ulcerative colitis and acute kidney rejection. The gene regulatory information allows the model to identify the biological regulators associated with the response, potentially giving mechanistic insight into differential clinical trial response. GRRANN [42], which was applied to the same data as creNET, uses a gene regulatory network to inform the structure of a neural network. Several other methods [43,44] have also used gene regulatory network structure to constrain the structure of a neural network, reducing the number of parameters to be fit and facilitating interpretation.

## Applications in genetics

Approaches that incorporate gene set or network structure into genetic studies have a long history [45,46]. Recent applications include expression quantitative trait loci (eQTL) mapping studies, which aim to identify associations between genetic variants and gene expression. netReg [47] implements a graph-regularized dual LASSO algorithm for eQTL mapping [48] in a publicly available R package. This model smoothens regression coefficients simultaneously based on networks describing associations between genes (target variables in the eQTL regression model) and between variants (predictors in the eQTL regression model). eQTL information is also used in conjunction with genetic variant information to predict phenotypes, in an approach known as Mendelian randomization (MR). In [49], a smoothing term derived from a gene regulatory network is used in an MR model. The model with the network smoothing term, applied to a human liver dataset, more robustly identifies genes that influence enzyme activity than a network-agnostic model. As genetic datasets grow, we expect that researchers will continue to develop models that leverage gene set and network databases.

## Other models incorporating biological structure

**Figure 3:**
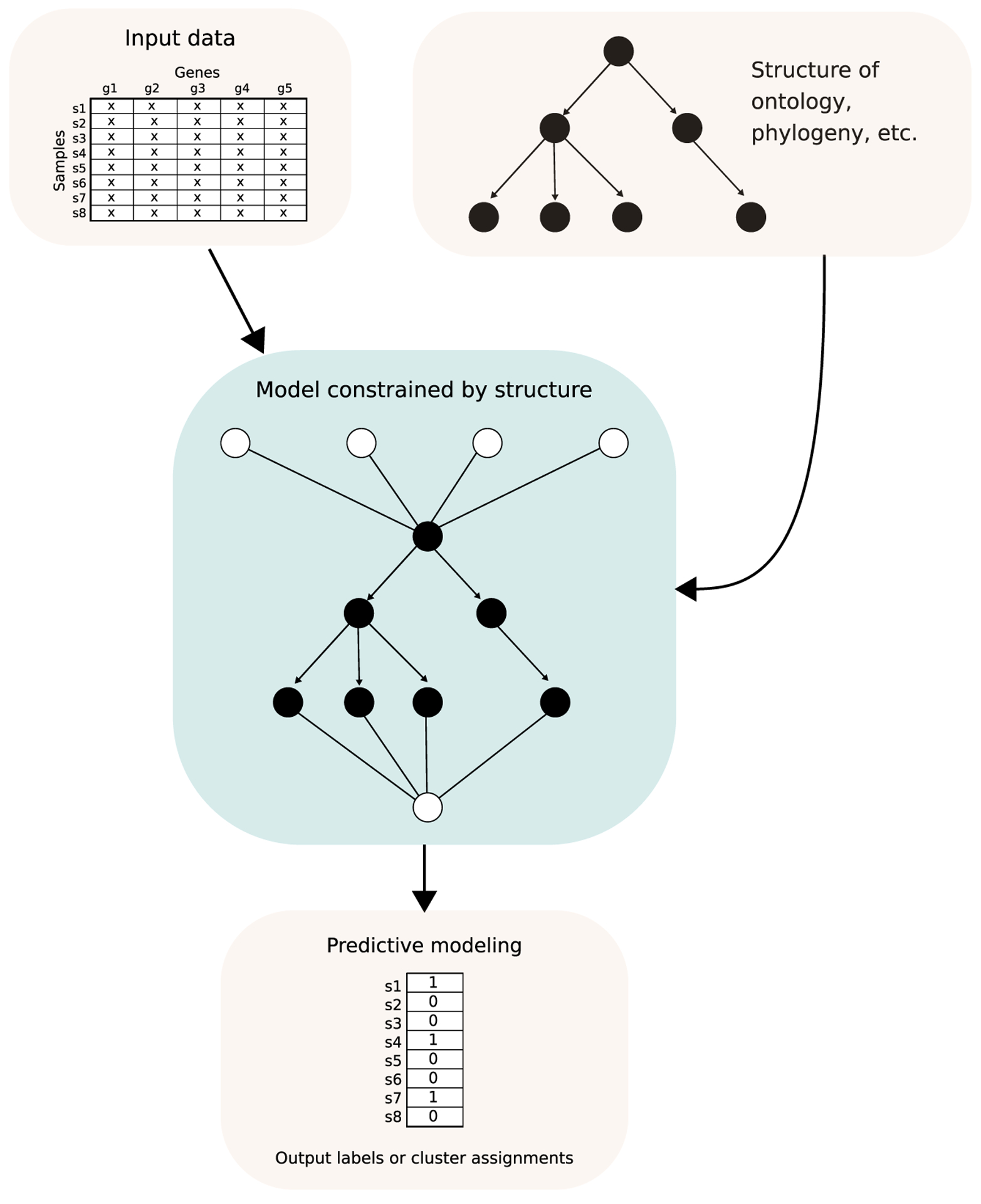
Directed graph-structured data, such as an ontology or phylogenetic tree, can be incorporated into machine learning models. Here, the connections in the neural network used to predict a set of labels parallel those in the tree graph. This type of constraint can also be useful in model interpretation: for example, if the nodes in the right tree branch have high neuron outputs for a given sample, then the subsystem encoded in the right branch of the tree graph is most likely important in making predictions for that sample. The ‘x’ values in the data table represent gene expression measurements.

Knowledge about biological entities is often organized in an ontology, which is a directed graph that encodes relationships between entities (see Figure 3 for a visual example). The Gene Ontology (GO) [3] describes the relationships between cellular subsystems and other attributes describing proteins or genes. DCell [50••] uses GO to inform the connectivity of a neural network predicting the effects of gene deletions on yeast growth. DCell performs comparably to an unconstrained neural network for this task. Additionally, it is easier to interpret: a cellular subsystem with high neuron outputs under a particular gene deletion can be inferred to be strongly affected by the gene deletion, providing a putative genotype-phenotype association. DeepGO [51•] uses a similar approach to predict protein function from amino acid sequence with a neural network constrained by the dependencies of GO. However, a follow-up paper by the same authors [52] showed that this hierarchy-aware approach can be outperformed by a hierarchy-naive CNN, which uses only amino acid sequence and similarity to labeled training set proteins. This suggests a trade-off between interpretability and predictive accuracy for protein function prediction.

Phylogenetic trees, or hierarchies describing the evolutionary relationships between species, can be useful for a similar purpose. glmmTree [53] uses a phylogenetic tree describing the relationship between microorganisms to improve predictions of age based on gut microbiome data. The same authors combine a similar phylogeny smoothing strategy with sparse regression to model caffeine intake and smoking status based on microbiome data [54]. Phylogenetic trees can also describe the relationships between subclones of a tumor, which are fundamental to understanding cancer evolution and development. Using a tumor phylogeny inferred from copy number aberration (CNA) sequencing data as a smoothing term for deconvolving tumor subclones provided more robust predictions than a phylogeny-free model [55]. The tree structure of the phylogeny and the subclone mixture model are fit jointly to the CNA data.

Depending on the application, other forms of structure or prior knowledge can inform predictions and interpretation of the model’s output. CYCLOPS [56] uses a circular node autoencoder [57] to order periodic gene expression data and estimate circadian rhythms. The authors validated the method by correctly ordering samples without temporal labels and identifying genes with known circadian expression. They then applied it to compare gene expression in normal and cancerous liver biopsies, identifying drug targets with circadian expression as candidates for chronotherapy. NetBiTE [58••] uses drug-gene interaction information from GDSC [59], in addition to protein interaction data, to build a tree ensemble model with splits that are biased toward high-confidence drug-gene interactions. The model predicts sensitivity to drugs that inhibit crucial signaling pathways in cancer, showing improved predictive performance compared to random forests, another commonly used tree ensemble model.

## Conclusions and future directions

As the quantity and richness of biomedical data have increased, sequence repositories and interaction databases have expanded and become more robust. This raises opportunities to integrate these resources into the structure of machine learning models. There have been several past attempts to benchmark and compare approaches to integrating structured data into predictive models in biomedicine, including the evaluation in Ref. [60] and more recent studies in Refs. [61] and [62]. Extending and broadening benchmarking efforts such as these will be vital, improving our understanding of the suitability of problem domains and datasets for the classes of methods described in this review.

Many methods described in this review have open-source implementations available; however, increased availability of performant and extensible implementations of these models and algorithms would facilitate further use and development. In the future, we foresee that incorporating structured biomedical data will become commonplace for improving model interpretability and boosting performance when sample size is limited.
